# Early prenatal diagnosis of thoraco-omphalopagus twins at ten weeks of gestation by ultrasound

**DOI:** 10.4274/tjod.89814

**Published:** 2016-06-15

**Authors:** Dilşat Herkiloğlu, Başak Baksu, Oya Pekin

**Affiliations:** 1 Zeynep Kamil Women’s and Children’s Training and Research Hospital, Clinic of Obstetrics and Gynecology, İstanbul, Turkey; 2 Zeynep Kamil Women’s and Children’s Training and Research Hospital, Clinic of Perinatology, İstanbul, Turkey

**Keywords:** monozygotic twins, conjoined twins, ultrasonography, Doppler ultrasonography

## Abstract

Early prenatal diagnosis of conjoined twins, an extreme form of monozygotic twinning, is very important for the further management and counselling of parents because they are associated with high perinatal mortality. We present a case of thoraco-omphalopagus twins diagnosed at ten weeks and four days of gestation by two-dimensional Doppler ultrasound, which was then terminated.

## PRECIS:

We present a case of thoraco-omphalopagus twins diagnosed at ten weeks and four days of gestation using two-dimensional doppler ultrasound.

## INTRODUCTION

Conjoined twins are a rare, complex complication seen in 1% of monochorionic twins that occurs with an estimated incidence of 1 in 50.000 to 1 in 200.000 live births^(1)^. Even though the degree and location of conjunction and the shared internal organs determine the prognosis of conjoined twins, they are associated with a high perinatal mortality rate; the overall survival rate is 25%. Although a smaller fraction born alive have anomalies incompatible with life, survivors need to have various correction operations because of many coexisting diseases^(1,2)^.

Therefore, early prenatal diagnosis of conjoined twins plays a crucial role in management and allows appropriate and timely counselling of the parents to decide among various options, which are (a) continuation of pregnancy with planned postnatal surgery, (b) termination of pregnancy, and (c) in cases of high-order multifetal pregnancies with a component of conjoined fetuses, multifetal pregnancy reduction or selective fetocide^(1)^.

In this report, we present a case of thoraco-omphalopagus twins diagnosed prenatally using two-dimensional (2-D) Doppler ultrasound (US) at ten weeks and four days of gestation.

## CASE REPORT

A gravida five, parity two, abortus two woman aged 32 years woman was referred to our perinatology clinic for first trimester screening with a combined test. Her obstetric history revealed two spontaneous vaginal deliveries and was unremarkable with respect to medication use or births with structural or chromosomal anomalies. She had no family history of multiple gestations.

**Figure 1:**
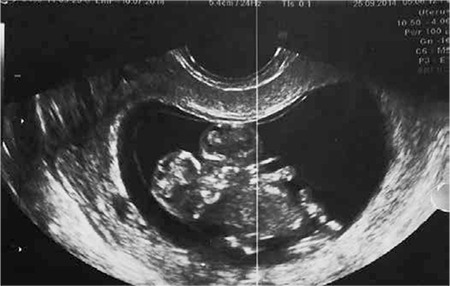
Ultrasound image

Transabdominal US scan using a 5 MHz probe with 2-D Doppler US (Voluson 730 PRO, GE Medical system) revealed a monochorionic monoamniotic twin pregnancy of 10 weeks and four days of gestation, according to her last menstrual date. One of the fetuses was anencephalic. Both fetuses had cystic hygroma. The fetuses were fused to each other at the chest (thoracopagus) and the umblicus (omphalopagus). Two upper and two lower extremities were seen for each fetus. There was only one heart beat. This was confirmed using a 5 MHz transvaginal probe. A diagnosis of conjoined twins was made using sonography (Figure 1).

**Figure 2:**
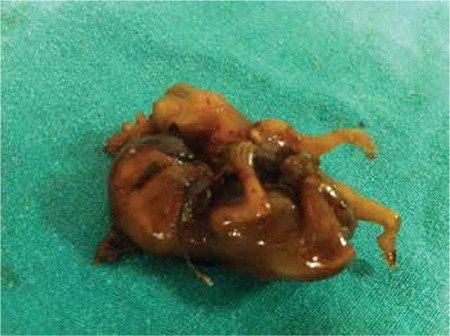
Macroscopic image of the induced abortion material

The couple was informed about the US findings and counseled about management options. They decided to terminate the pregnancy. The induced abortion material was sent for pathogic examination. The result was conjoined twins with two bodies fused from the upper thorax to lower belly (Figure 2). Both fetuses were female. Two upper and two lower extremities were seen for each fetus. The pathology report revealed one heart, one liver, two stomachs, and two kidneys shared by the twins. Therefore, the ultrasonographic diagnosis of conjoined twins of thoraco-omphalopagus type was confirmed.

## DISCUSSION

Conjoined twins is an extreme form of monozygotic twinning. Incomplete fusion of a single zygote at the primitive streak stage (15-17 days) during blastogenesis is considered to be responsible for this condition^(3)^. Therefore, when there is a case of monochorionic-monoamniotic pregnancy, the possibility of conjoined twins should always be kept in mind.

Sonographic characteristics that raise the suspicion of conjoined fetuses are polyhydramnios seen in 50-76% of cases, bi-breech and face-to-face presentation of twins^(4)^. Moreover, for early diagnosis of conjoined twins, specific ultrasound features have been proposed and reports of diagnosis as early as seven weeks and six days of gestation have been published. However, it is still possible to miss or misdiagnose conjoined twins beacuse of various misleading sonographic signs^(5,6)^. Increased nuchal translucency (NT) was observed in six fetuses of four conjoined twins. In the present case, both fetuses had cyctic hygromas. This observation highlights the importance of increased NT in multifetal pregnancies during the 11-14 weeks scan by raising the possibility of conjoined twins so that a more careful examination should be considered^(7)^. Therefore, the 11-14 weeks 2-D Doppler US scan remains the mainstay of prenatal diagnosis of conjoined twins^(1)^. This was also the diagnostic method used in the prenatal diagnosis in this case report. Moreover, very early diagnosis seems not to add any practical information compared with detection in the 11-14 weeks scan because either repeat US, magnetic resonance imaging (MRI) or 3-D US had to be used to confirm the diagnosis of conjoined twins in such reports^(8)^. It must be stressed that even though ultrasonographic features such as an absent separating membrane in monochorionic twins, bifid appearance of the fetal pole, and inseparable bodies or heads despite fetal movements suggest the diagnosis, the 11-14 week scan with 2-D Doppler US is still the diagnostic imaging modality in these cases^(9)^. However, ultrasound does have pitfalls; therefore, modern imaging modalities like 3-D US, 4-D US or fetal rapid MRI can be useful to overcome these disadvantages in the diagnosis of conjoined twins. In addition, if the parents decide to continue the pregnancy, MRI is used to determine the site and percentage of conjunction to further predict the success of surgical seperation and postnatal prognosis.

Conjoined twins are very rarely seen in human pregnancies. However, they should be kept in mind when monochorionic twins are being examined during the 11-14 week scan, especially when increased NT is observed because early and accurate prenatal recognition is essential for parental counseling to decide for or against continuation of pregnancy. If parents decide to continue the pregnancy, prenatal surveillance and postnatal management should be planned. The obstetricians role in prenatal diagnosis, counseling, and organization of interdiciplinary medical care is indispensible in cases of conjoined twin.
